# Professor Mark Wainberg

**DOI:** 10.1186/s12977-017-0353-6

**Published:** 2017-05-05

**Authors:** Monsef Benkirane, Ben Berkhout, Persephone Borrow, Ariberto Fassati, Masahiro Fujii, J. Victor Garcia, Paul Gorry, Andrew Lever, Johnson Mak, Monique Nijhuis, Klaus Strebel, Francois Venter, Robin Weiss

**Affiliations:** Editorial Board, London, UK

When Kuan-Teh Jeang (‘Teh’ to everyone) made the bold and prescient decision to join the earliest wave of pure online academic publishing and founded the journal Retrovirology, Mark Wainberg was one of the simplest and most obvious choices for him to invite to join the editorial team. Mark’s long established and highly respected position in the field of HIV and AIDS research added enormously to the embryonic journal’s immediate credibility and stature. Mark’s seminal achievements in recent years have been in antiretroviral therapy and viral resistance mechanisms but, in a publishing career on HIV spanning 30 years and over 550 publications, there were few areas of HIV research that he did not investigate and he brought that enormous range of expertise and experience to Retrovirology. His many achievements in the field will be described in detail by others, including his trainees and colleagues from Canada, in a shortly to be published obituary in this journal. When Teh himself was so sadly taken from us it was again Mark’s stature and reputation and his boundless enthusiasm and energy that was so important in maintaining the momentum and profile of the Journal as he took on the role of Co-Editor in Chief.

Mark was concerned with all aspects of conquering HIV/AIDS, from public health approaches through to fundamental molecular biology, vaccinology and pharmacology and he was an outspoken advocate for research on HIV and for maintaining public awareness of the AIDS epidemic. These he expressed in his scientific papers, editorials and reviews, many of which appeared in Retrovirology. Mark was a powerful supporter of the Frontiers in Retrovirology conferences that made such an important contribution to the journal’s profile.

In recent months there have been important decisions made at the journal to publish thematic reviews on specific contemporary issues in retrovirus research and the first of these are already commissioned. Similarly a decision was made to expand the remit of the journal to include more clinical research aspects and to expand our geographical reach. To that end we have been fortunate to recruit Professor Francois Venter from South Africa to the Editorial team. Mark’s contribution to and support for these exciting developments was crucial. It is a sad irony that he will not see the benefits of these innovations but they will be part of his legacy and a part of the enormous contribution he has made to Retrovirology in its first years. He will be sadly missed by all of the editorial team.

